# Porto‐Pulmonary Hypertension in Children: Insights From a National Registry

**DOI:** 10.1002/pul2.70133

**Published:** 2025-09-24

**Authors:** Sadia Quyam, Alastair Baker, Alistair Calder, Shahin Moledina

**Affiliations:** ^1^ UK Service for Pulmonary Hypertension in Children Great Ormond Street Hospital for Children London UK; ^2^ Institute of Cardiovascular Sciences University College London London UK; ^3^ Paediatric Liver Centre King's College Hospital London UK

**Keywords:** pediatric liver disease, pediatric pulmonary hypertension, porto‐systemic shunts, pulmonary arterial hypertension

## Abstract

Porto‐pulmonary hypertension (PoPH) represents a rare but significant form of pulmonary arterial hypertension (PAH) in children. Despite its clinical importance, systematic analyses of paediatric presentations and outcomes remain limited. We analysed the United Kingdom National Registry for Paediatric Pulmonary Hypertension (2001–2022) identifying children with PoPH through standardised diagnostic criteria including cardiac catheterisation and cross‐sectional imaging. In our cohort of 12 patients (58% female, median age 4 years, range: 3 months–12 years), congenital porto‐systemic shunts (CPSS) were the predominant pathology (58%). We found a high prevalence of genetic abnormalities (50%) and congenital heart disease (50%). Haemodynamic assessment revealed evidence of pulmonary vascular disease (mean pulmonary artery pressure 38 mmHg, range 20–52 mmHg; mean pulmonary vascular resistance index 6.1 WU·m², range 4.2–9.0 WU·m²) without vaso‐reactivity. Over a median follow‐up of 8.2 years, three patients achieved resolution of pulmonary hypertension after definitive treatment of underlying liver pathology. Four deaths occurred during follow‐up: three from progressive PAH and one unrelated death that occurred 2 years following PAH resolution. Our analysis reveals distinctive features of paediatric PoPH, including predominant CPSS aetiology, and earlier age of onset than previously reported. Multi‐modality imaging proved essential for diagnosis, as initial ultrasound missed CPSS in 5/7 cases. The variable treatment outcomes emphasise the importance of individualised therapeutic approaches and sustained clinical surveillance.

## 1 Introduction

Porto‐pulmonary hypertension (PoPH) is a subtype of pulmonary arterial hypertension (PAH), occurring in association with portal hypertension or portal venous abnormalities [1]. Current diagnostic criteria for paediatric PAH require a mean pulmonary arterial pressure > 20 mmHg at rest, pulmonary arterial wedge pressure ≤ 15 mmHg, and pulmonary vascular resistance index ≥ 3 WU·m² [1]. In congenital porto‐systemic shunts (CPSS), portal pressures may be normal; however, these cases are classified as PoPH because portal venous system abnormalities drive pulmonary vascular remodelling [1].

The epidemiology of PoPH differs markedly between adults and children. Adult PoPH predominantly occurs in cirrhotic liver disease, affecting 0.7%–5% of these patients [2, 3, 4, 5]. In paediatric populations, PoPH more commonly arises from congenital vascular anomalies. While rare overall, it affects 0.5%–0.9% of children with chronic liver disease or awaiting liver transplantation [6, 7] and 6%–13% of those with CPSS [8, 9, 10, 11].

Analysis of national registries in Spain [11] and the United Kingdom [12], along with the North American multi‐centre PPHNet registry [13], suggests PoPH represents 0.4%–1.3% of paediatric pulmonary hypertension (PH) cases and 1%–2% of paediatric PAH cases. Our current understanding of disease phenotypes and outcomes is limited by the small number of reported cases [10, 14, 15, 16] (Table 1).

**Table 1:** Previously published paediatric porto‐pulmonary hypertension cohorts (2005‐2021).

Study (Year)	*N*	Age^a^	Primary Aetiology	Baseline Parameters	Treatment (*n*)	Outcomes: Deaths/Survived	Follow‐up (mo)
Bobhate (2021)	10	12.5 (4–18)	CPSS (10)	mPAP: 38.7 ± 8.4; PVRI: 6.8 ± 2.1	NR	1/8^b^ (4/6 CPSS closure resolved)	6 (3–12)
Joye (2021)	6	13 (10–15)	CPSS (3); BA (1); CTPV (1); Cirrhosis (1)	mPAP: 47 (32–70); PVR: 6.6 (4.3–15.4)	Mono (1); Combo (5)	1/5 (1 resolution post CPSS closure)	39 (6–48)
Tingo (2017)	5	14 (8–17)	CPSS (2) BA (1); PVT (1); unknown (1)	mPAP: 49 ± 12.3; PVRI: 8.2 ± 3.4	Mono (2); Combo (3)	3/2 (0 resolution)	NR
Condino (2005)	7	13.8 (0.6–17)	BA (3); CTPV (2); Other (2)	mPAP: 65 ± 20.0; PVRI: 12.4 ± 8.6	CCB (3); Mono (1); Epo (3)	4/3 (0 resolution)	NR

Abbreviations: BA = biliary atresia, CCB = calcium channel blockers, Combo = combination therapy, CPSS = congenital porto‐systemic shunt, CTPV = cavernous transformation of portal vein, Epo = epoprostenol, mPAP = mean pulmonary artery pressure (mmHg), Mono = monotherapy, NR = not reported, PVRI = pulmonary vascular resistance index (WU·m²), PVR = pulmonary vascular resistance (WU), PVT = portal vein thrombosis.

^a^ Age presented as median (range) or mean ± SD in years.

^b^ One patient lost to follow‐up.

The pathophysiology is thought to centre on the consequences of porto‐systemic shunting, whether through discrete congenital vessels or networks of small collateral vessels developing in portal hypertension [17]. Evidence suggests this altered circulation exposes the pulmonary vasculature to two key pathogenic mechanisms: increased pulmonary blood flow with elevated wall shear stress; and direct exposure to vasoactive mediators that normally undergo hepatic first‐pass metabolism [18, 19]. While these mechanical and biochemical factors likely contribute to progressive vascular remodelling [17], the relative importance of each mechanism and other potential factors remains incompletely understood. Understanding the clinical manifestations and outcomes across different patient populations may provide insights into these underlying mechanisms.

Recent evidence from published case series [14, 16] suggests that paediatric PoPH may represent a distinct clinical entity from adult disease. However, recognition remains challenging due to subtle clinical manifestations and technical limitations of conventional imaging in detecting CPSS.

The UK National Registry for Paediatric Pulmonary Hypertension provides a unique opportunity to examine this rare condition through standardised, population‐based data collection. This study analyses registry data to characterise the clinical phenotypes, diagnostic features, and outcomes of paediatric PoPH.

## 2 Methods

### 2.1 Study Design and Population

This retrospective analysis included all patients diagnosed with PoPH between January 1, 2001 and January 1, 2022 in the UK National Registry for Paediatric Pulmonary Hypertension. The registry encompasses comprehensive clinical data for all children (< 18 years) diagnosed with PH within the UK national service.

### 2.2 Diagnostic Criteria

PoPH diagnosis required two elements: confirmed PH and evidence of liver cirrhosis, CPSS, or portal vein abnormalities with porto‐systemic shunting. PH was confirmed through cardiac catheterisation or noninvasive assessment with supportive echocardiographic signs, as validated by a PH multi‐disciplinary team. Alternative causes were excluded through a comprehensive evaluation including chest CT, autoimmune screening, lung function testing. Cardiac magnetic resonance imaging (MRI) was performed in children who could tolerate the procedure without general aneasthesia. Disease resolution was defined as normalisation of pulmonary arterial pressures on right heart catheterisation or noninvasive imaging after withdrawal of PH‐specific therapy.

### 2.3 Data Collection

We extracted demographic, clinical, haemodynamic, treatment and follow‐up data from electronic medical records.

## 3 Results

### 3.1 Patient Characteristics

In our cohort of 12 patients with PoPH, 7 (58%) were female, all were born at ≥ 36 weeks' gestation with median age at diagnosis of 4 years (range 3 months—12 years) (Table 2). These 12 cases represent approximately 1.1% of the 1101 children with confirmed PH in the UK National Paediatric Pulmonary Hypertension Service registry [12]. CPSS was the predominant aetiology (*n *= 7, 58%), followed by cavernous transformation of the portal vein (*n* = 2), liver cirrhosis (*n* = 2) and hepatic veno‐occlusive disease (*n* = 1). PH diagnosis preceded identification of hepatic disease in 75% of cases.

**Table 2:** Baseline characteristics and outcomes by patient group.

	Overall (*n* = 12)	CPSS (*n* = 7)	Other (*n* = 5)
Female, *n* (%)	7 (58)	5 (71)	2 (40)
Age at PH diagnosis, years	4 (0–13)	7 (1–13)	2 (0–12)
CPSS	7 (58)		
CTPV	2 (16)		
Cirrhosis	2 (16)		
LVOD	1 (8)		
CHD	6 (50)	4 (57)	2 (40)
Genetic diagnosis	6 (50)	4 (57)	2 (40)
WHO functional class			
I	2 (16.6)	1 (14.2)	1 (20)
II	3 (25.0)	2 (28.6)	1 (20)
III	4 (33.3)	2 (28.6)	2 (40)
IV	3 (25.0)	2 (28.6)	1 (20)
Catheterisation	10 (83)	6 (86)	4 (80)
mPAP, mmHg	39 (20–52)	39 (20–52)	38 (28–48)
RAP, mmHg	7 (3–10)	6 (3–9)	8 (5–10)
PVRI, WU·m²	6.1 (4.2–9.0)	5.3 (4.2–9.0)	6.7 (5.5–7.8)
CI, L/min/m²	4.0 (2.6–6)	4.1 (2.7–5.5)	4.5 (2.6–6.4)
MRI	8 (66)	6 (86)	2 (40)
LVEF, %	62 (55–70)	63 (56–70)	61 (55–66)
LVCI, L/min/m²	4.4 (2.6–6.5)	4.5 (2.6–6.5)	4.0 (3.6–4.3)
RVEF, %	48 (25–58)	51 (47–58)	41 (25–56)
RVEDVi, L/min/m²	117 (54–239)	92 (54–123)	179 (118–239)
Death	4 (33)	1 (8)	3 (60)
Liver transplant	3 (25)	1 (8)	2 (40)
PH Resolution	3 (25)	1 (8)	2 (40)

*Note:* Data presented as median (range) or *n* (%) unless otherwise specified.

Abbreviations: CI = cardiac index, CHD = congenital heart disease, CPSS = congenital portosystemic shunts, CTPV = cavernous transformation of the portal vein, L/RVEF = left/right ventricular ejection fraction, LVCI = left ventricular cardiac index, mPAP = mean pulmonary artery pressure, RAP = right atrial pressure, PH = pulmonary hypertension, PVRI = pulmonary vascular resistance index, RVEDVi = right ventricular end‐diastolic volume index.

### 3.2 Genetic and Cardiac Associations

Genetic abnormalities were present in six patients (50%) including Down syndrome, Adams‐Oliver syndrome, heterozygous MTHFR C677T mutation, heterozygous variant of uncertain significance in the EI2AK4 gene, and a copy number variation involving 1p deletion and 17q gain. One additional patient had an undiagnosed dysmorphic syndrome. Congenital heart disease (CHD) occurred in six patients (50%). Predominantly atrial septal defects (*n* = 5, three with additional defects) (Table 3). Four CHD cases demonstrated bidirectional shunting and one showed purely right to left flow at PH diagnosis. While disorders of laterality are known to be associated with portal vein anomalies, none were present in our cohort.

**Table 3:** Characteristics of congenital heart defects.

Case	CHD type	Status at PH diagnosis	Shunt direction at PH diagnosis	Assessment of CHD‐ PH relationship	Liver diagnosis
1	VSD + ASD + PDA	All surgically closed 5 year before PH diagnosis	N/A	Minimal ‐ tiny residual ASD	CPSS
2	ASD	Unrepaired at diagnosis, later underwent fenestrated closure	Left to right	Partial: Qp:Qs 1.6:1 early repair. Insufficent to explain severity of PH	CPSS
3	ASD + VSD	Unrepaired, presented at 1 month of age	Left to right	Moderate: flow contribution but concurrent with CPSS/AVM diagnosis	CPSS with large hepatic AVMs
4	ASD + VSD	Unrepaired	Bidirectional	Partial ‐ Qp:Qs 1.4:1	CPSS
5	ASD	Unrepaired	Bidirectional	Minimal—small defect	Cryptogenic liver cirrhosis
6	ToF with absent pulmonary valve	ToF repaired before PH diagnosis	N/A	Minimal—no residual defect. Oesphageal varices developed 1 year post repair	CTPV

*Note:* CHD type describes the specific cardiac defects; Status details the condition of these defects at PH diagnosis; Shunt direction indicates blood flow patterns observed at diagnosis on echo; Assessment evaluates the assessed contribution of CHD to PH based on timing, shunt magnitude, and clinical presentation; Liver diagnosis identifies the underlying hepatic/portal abnormality.

Abbreviations: ASD = atrial septal defect, AVM = arteriovenous malformation, CPSS = congenital porto‐systemic shunt, CTPV = cavernous transformation of portal vein, PDA = patent ductus arteriosus, Qp:QS = pulmonary to systemic flow ratio, ToF = Tetralogy of Fallot, VSD = ventricular septal defect.

### 3.3 Clinical Presentation and Initial Assessment

Presentations ranged from incidental detection (*n* = 4) to symptomatic manifestations including exercise intolerance (*n* = 4), unexplained oxygen requirement (*n* = 3), and syncope (*n* = 1). At presentation, seven patients (58%) had severe functional limitation (WHO Functional Class III: *n* = 4, 33% and Class IV: *n* = 3, 25%). Cardiac catheterisation (*n* = 10, 83%), demonstrated mean pulmonary artery pressure (mPAP) of 38 mmHg (range 20–52 mmHg), mean cardiac index 4.4 L/min/m^2^ (range 2.6–6.4 L/min/m^2^) and mean pulmonary vascular resistance index (PVRI) of 6.1 WU·m² (range 4.2–9.0 WU·m²). None of the eight patients who underwent acute vaso‐reactivity testing showed positive responses.

### 3.4 Imaging Findings

Cardiac MRI (*n* = 7) showed preserved right ventricular (RV) function in most patients, with a mean RV ejection fraction of 48% (range 25%–58%). Only one patient had significant RV dysfunction. The mean cardiac index on cardiac MRI was 5.4 L/min/m² (range 2.6–8.4 L/min/m²) with elevated values (> 4 L/min/m²) in five patients, including three with CPSS.

The diagnostic pathway for identifying CPSS varied among patients. Initial ultrasound evaluation identified two cases ‐ one known before PH diagnosis and one complex hepatic arteriovenous malformation with patent ductus venosus (classified as CPSS due to associated porto‐systemic shunting). Five cases were initially missed on standard ultrasound: two were subsequently identified on CT during cardio‐pulmonary assessment, one during cardiac MRI, and two on repeat targeted ultrasound examination. Importantly, in four of the five initially missed cases, the shunts could be visualised on targeted ultrasound once the anatomical location was known.

The anatomical distribution of CPSS (Table 4) included four Abernethy malformations (one type 1b with a superior mesenteric vein to right atrial connection; three type 2 with varying venous connections) two Park type 1 intrahepatic shunts to the IVC and one complex hepatic arteriovenous malformation with patent ductus venosus.

**Table 4:** Anatomical and clinical features of congenital porto‐systemic shunts (*n* = 7).

Case	Shunt anatomy	Liver disease	Location/Classification	Closure status	Status at last follow‐up
**1**	SMV‐RA	Yes—FNH	Central, Abernethy 1b	Complete (liver transplant)	PH resolved (confirmed by cardiac catheterisation)
**2**	PV‐left renal	No	Peripheral, Abernethy 2	Complete	Improved but persistent PH (TR 3.5 m/s on dual oral therapy 9 years post closure)
**3**	Spleno‐renal	No	Peripheral, Abernethy 2	Incomplete (tiny remnant)	Improved (No TR, PR EDV 2.5 m/s on monotherapy)
**4**	SMV/SV to IVC	Yes—FNH	Central, Abernethy 2	Not attempted	Stable (TR 3 m/s on triple therapy)
**5**	Park type 1 CIPS	Yes	Central	Complete	Improved (TR 2.7 m/s on monotherapy)
**6**	Park type 1 CIPS	Yes	Central	Not attempted	Stable (TR 2.7 m/s on dual therapy)
**7**	Hepatic AVM with Patent DV	Yes	Central	Incomplete	Died (rapidly progressive PAH)

Abbreviations: AVM = arteriovenous malformation, CIPS = congenital intrahepatic porto‐systemic shunt, DV = ductus venosus, FNH = focal nodular hyperplasia, IVC = inferior vena cava, PV = portal vein, RA = right atrium, SMV = superior mesenteric vein, SV = splenic vein.

### 3.5 Treatment and Outcomes

Ten patients (83%) received PH‐specific therapy—eight as monotherapy (phosphodiesterase‐5 inhibitors or endothelin receptor antagonists), one as dual therapy, and one with additional inhaled prostacylin analogue. Two patients did not receive PH‐specific therapy; both had undergone liver transplantation and demonstrated a trajectory of improvement during follow‐up.

Five CPSS patients underwent intervention: one liver transplantation and four shunt closures. There was no procedure‐related mortality. Following intervention, one patient (post‐transplant) achieved PH resolution confirmed by cardiac catheterisation. Three patients showed improvement in noninvasive markers of PH while continuing pre‐intervention PH‐specific therapy. One patient with complex hepatic AVMs had progressive PAH despite multiple embolisation attempts, as the vessels repeatedly reopened, resulting in persistent shunting and ultimately death.

During median follow‐up of 8.2 years (range 12 days–14 years) three patients achieved PH resolution: two after liver transplantation, and one following defibrotide treatment for hepatic veno‐occlusive disease. Resolution was confirmed by cardiac catheterisation in one transplant patient and assumed by echocardiography in the remaining two patients.

Four patients died, three from progressive PAH. One death, unrelated to PoPH, occurred 2 years after resolution of PH post‐liver transplant. The three PAH‐related deaths occurred in female patients who were younger at diagnosis (median age 1 year vs. 9 years in survivors) had genetic abnormalities and presented in advanced WHO functional class (III–IV), compared to only 51% of survivors presenting in these classes.

### 3.6 Details of Fatal Cases

A neonate with Down syndrome and cardiac shunts (atrial septal defect and multiple ventricular septal defects with bidirectional flow) presented with significant respiratory distress at birth. Initial management targeted presumed persistent pulmonary hypertension of the newborn. Further evaluation identified extensive porto‐systemic shunting: a large patent ductus venosus and multiple aorto‐portal shunts converging on a large portal venous aneurysm. Despite multiple interventional shunt occlusion procedures (three procedures over 2 months), complete occlusion could not be achieved. Postprocedure angiography consistently demonstrated residual shunting and follow‐up ultrasound confirmed re‐opening of previously treated vessels. Despite these attempts, PAH progressed rapidly. Intensive care was withdrawn at 4 months of age. Post‐mortem histology demonstrated advanced pulmonary vascular disease with intimal fibrosis.

A 7‐month‐old infant developed PAH following liver transplantation at 3 months of age for end‐stage liver disease of unknown aetiology with established portal hypertension (gastric varices, splenomegaly, nodular liver). Early graft dysfunction with rising transaminases occurred post‐transplant. Cardiac catheterisation confirmed PAH. Despite initiation of sildenafil, clinical deterioration progressed rapidly, culminating in cardiac arrest 5 months later. The diagnosis remained uncertain given the complex post‐transplant course, with differentials including POPH, VOD, and mitochondrial disease.

A 10‐year‐old presented with Adams Oliver syndrome, Tetralogy of Fallot with absent pulmonary valve, and portal vein cavernoma. Treatment was limited to bosentan monotherapy due to high bleeding risk from thrombocytopenia, high INR and known oesophageal varices. Attempted Meso‐Rex procedure was unsuccessful due to aplasia/hypoplasia of the intra‐hepatic portal venous system. The patient subsequently developed progressive right ventricular failure.

## 4 Discussion

This analysis of the UK National Registry for Paediatric PH reveals three key characteristics of paediatric PoPH: a phenotype distinct from adult disease, specific diagnostic challenges, and heterogenous treatment outcomes.

### 4.1 Insights From Combined Literature

When our cohort (*n* = 12) is considered alongside cases from previously published paediatric series (*n* = 28, summarised in Table 1) the combined literature describes 40 paediatric cases of PoPH. Analysis of this aggregated cohort reveals important aetiological patterns: CPSS as the predominant aetiology (22/40, 55%, including 7 from our cohort), followed by biliary atresia (5/40, 13%, all from previous reports), and CTPV (5/40, 12.5%, including 2 from our cohort).

Treatment outcomes appear to differ by underlying pathology, with mortality in CPSS at 9% (2/22), compared to 60% (3/5) in biliary atresia and CTPV, suggesting that CPSS‐associated PoPH may represent a more favourable prognostic subgroup, potentially due to the technical feasibility of definitive anatomical correction and preserved hepatocellular function.

Across five liver transplantations reported across the literature (three from our cohort, two from Joye et al. [16]). Outcomes were highly variable and influenced by complex case‐specific factors, Resolution of PH occurred in 2/5 (40%) post liver transplantation. The cases from Joye et al. demonstrated complexity—one patient had an underlying MC4R mutation leading to left heart disease with persistent PH progression, while the second (biliary atresia) showed clinical improvement but residual PAH on triple therapy. This heterogeity suggests liver transplantation outcomes in PoPH may be influenced by underlying aetiology, co‐morbidities, timing of intervention and pre‐existing pulmonary vascular disease severity. While it is not possible to draw conclusions about efficacy from such small numbers, these cases highlight the importance of comprehensive assessment and individualised decision‐making.

### 4.2 Diagnostic Challenges and Clinical Phenotype

The median age at diagnosis in our cohort was 4 years, compared to 12.5–14 years in earlier series [14, 15, 16], suggesting earlier manifestation of PoPH in childhood than previously recognised. The predominance of CPSS (58%) over cirrhotic liver disease, coupled with a high prevalence of genetic abnormalities (50%), suggests that paediatric PoPH may primarily represent a developmental disorder rather than merely a complication of portal hypertension.

A clinically significant observation was that PH diagnosis preceded identification of hepatic disease in 75% of cases. Initial abdominal ultrasonography failed to identify CPSS in five cases, though these abnormalities were subsequently visible on targeted examination after identification on cross‐sectional imaging. This diagnostic sequence, where PH is recognised before hepatic disease, has been reported in previous series [14, 15], highlighting the importance of systematic portal evaluation in children presenting with unexplained PH.

We propose a stepwise evaluation beginning with dedicated abdominal ultrasonography targeting common shunt locations, particularly left renal vein connections, which can be difficult to visualise. Direct communication with the sonographer about clinical suspicion of CPSS is essential to improve diagnostic yield. When ultrasound findings are equivocal or incomplete in the context of unexplained PH, additional cross‐sectional imaging should be considered. Either contrast‐enhanced CT (with portal venous phase imaging) or magnetic resonance angiography. In some cases, these modalities can be combined with cardiac evaluation. Following identification of portal venous abnormalities on any imaging modality, correlation with targeted ultrasound is valuable for confirmation and enhancing future diagnostic capabilities.

A notable clinical observation was that among the seven patients in WHO FC III/IV, the majority had either normal/mildly impaired RV function assessed by either echocardiography or MRI. This apparent disconnect between functional class and RV function suggests symptom burden may be influenced by additional factors such as comorbidities, extracardiac manifestations of complex multi‐system disorders, or disease‐specific metabolic derangements. This finding further emphasises the importance of comprehensive evaluation beyond cardiac imaging when assessing disease severity in paediatric PoPH.

### 4.3 Prognostic Factors and Mechanisms of Resolution

Our cohort revealed patterns that may inform risk stratification and treatment selection. Fatal cases were characterised by younger presentation (median age 1 year vs. 9 years in survivors), female predominance, and advanced WHO Functional Class at diagnosis. The presence of genetic abnormalities in all deceased patients suggests that developmental factors may influence disease severity and progression.

This variable response to intervention across the combined literature highlights the multifactorial nature of disease resolution. Haemodynamic assessment at diagnosis consistently demonstrated established pulmonary vascular disease, with elevated pulmonary vascular resistance (PVR) and absence of vaso‐reactivity in all tested patients. Of our 12 patients, three achieved complete resolution through distinct therapeutic mechanisms: liver transplantation without targeted PAH therapy for two patients (one with CPSS and focal nodular hyperplasia, one with cirrhosis) and defibrotide treatment for hepatic veno‐occlusive disease in the third.

This suggests that restoration of normal liver function, particularly detoxification capacity, may be important for pulmonary vascular homeostasis. This clinical observation aligns with emerging understanding of the liver's role in producing factors that regulate pulmonary vascular tone and remodelling. For example, bone morphogenetic protein 9 (BMP9), primarily synthesised in the liver, is increasingly recognised as an important regulator of pulmonary vascular tone and remodelling [20]. Disruptions in hepatic production or signalling of such factors could contribute to pulmonary vascular disease in the context of liver dysfunction or porto‐systemic shunting.

Analysis of CPSS patients across the combined literature [15, 16] revealed that complete elimination of porto‐systemic shunting, whether through anatomical closure (40% achieving resolution from 15 attempted closures) or liver transplantation, yielded better outcomes than partial correction. This observation supports the potential reversibility of pulmonary vascular remodelling when the underlying pathophysiological driver is fully addressed. However, the heterogeneity in treatment responses, even among patients with similar underlying pathologies, highlights the need for individualized therapeutic approaches guided by comprehensive assessment of both hepatic and pulmonary vascular pathology.

## 5 Conclusion

This national registry analysis supports three conclusions: First, paediatric PoPH represents a distinct clinical entity from adult disease, characterised by earlier onset (median age 4 years vs. 12.5–14 years in earlier series), predominant CPSS aetiology, and frequent genetic associations. Second, accurate diagnosis requires systematic multi‐modality imaging, as initial ultrasound alone may miss portal venous abnormalities. Finally, while complete elimination of porto‐systemic shunting can lead to disease resolution in some cases, early age at presentation and genetic abnormalities may comprise a high‐risk group requiring particularly vigilant monitoring and management.

## 6 Study Limitations

We acknowledge limitations inherent to registry‐based research, including the retrospective nature of data collection and potential referral bias. The rarity of the condition resulted in a small sample size which may limit generalisability.

## Author Contributions

S.M. and S.Q. conceptualised the study. A.C. analysed the imaging. S.Q. performed data curation and analysis. S.Q., S.M., and A.B. wrote and revised the original draft. The final manuscript was reviewed by S.Q., S.M., A.B., and A.C.

## Ethics Statement

This study was conducted in accordance with Declaration of Helsinki under ethics committee approval 17/LO/0008 with individual informed consent waived for anonymised data analysis.

## Conflicts of Interest

S.Q., A.C. and A.B. have nothing to declare. S.M. has acted as a consultant for Janssen‐Cilag ltd and GSK.

## Guarantor Statement

Dr. Sadia Quyam is the guarantor of this study, had full access to all study data, and takes responsibility for the integrity of the data and accuracy of the data analysis.
